# SARS-CoV-2 Exposure Elicits a Strong Mucosal Antibody Response That May Facilitate Quicker Mucosal Viral Clearance

**DOI:** 10.3390/v18091034

**Published:** 2026-09-17

**Authors:** Muruganantham Lillimary Eniya, Shervin Dokht Sadeghi Nasab, Albert Judith, Frederick Clasen, Beulah Faith, Selvamuthu Poongulali, Jayaraman Bhagavad Gita, Chakrapani Ashok, Velmurugan Raghavi, Subramanian Vedavalli, Chandra Lavanya, Kannan Ranganathan, Gunaseelan Rajan, Nagalingeswaran Kumarasamy, David Moyes, Mark Ide, Saeed Shoaie, Yuko Kurushima, Daljit Jagdev, Newell Johnson, Stephen Challacombe, Priya Kannian

**Affiliations:** 1 VHS Laboratory Services, Department of Clinical Research, The Voluntary Health Services, Chennai 600113, India; enianand98@gmail.com (M.L.E.); judithalbert1998@gmail.com (A.J.); beulah@cartcrs.org (B.F.); poongulali@cartcrs.org (S.P.); kumarasamy@cartcrs.org (N.K.); 2 Centre for Host Microbiome Interactions, Faculty of Dentistry, Oral & Craniofacial Sciences, Floor 17, Guy’s Hospital Tower, King’s College London, London SE1 9RT, UK; shervin_dokht.sadeghi_nasab@kcl.ac.uk (S.D.S.N.); frederick.clasen@kcl.ac.uk (F.C.); david.moyes@kcl.ac.uk (D.M.); mark.ide@kcl.ac.uk (M.I.); saeed.shoaie@kcl.ac.uk (S.S.); yuko.kurushima@kcl.ac.uk (Y.K.); dkjagdev@yahoo.co.uk (D.J.); n.johnson@griffith.edu.au (N.J.); 3 Chennai Dental Research Foundation, Chennai 600004, India; gita70.geetha@gmail.com (J.B.G.); ashok.chakrapani@gmai.com (C.A.); dental.cdrf@gmail.com (V.R.); drvedavalli@rajandental.com (S.V.); drgunaseelan@rajandental.com (G.R.); 4 Ragas Dental College and Hospital, Chennai 600119, India; lavanyac2210@yahoo.com (C.L.); ranjay22@gmail.com (K.R.); 5 Griffith University Dental School, Southport, QLD 4215, Australia

**Keywords:** SARS-CoV2, oral fluid, mucosal antibodies, IgA, IgG, SIgA, avidity

## Abstract

The entry of SARS-CoV-2 via the mucosal surfaces of the upper respiratory tract, including the oral cavity, may be influenced by innate and adaptive immunity. We aimed to determine whether antibodies in secretions might influence the SARS-CoV-2 burden and thus the severity of infection. Blood and stimulated oral fluid (SOF) samples were collected at recruitment (d0) from 173 participants and sequentially at d14, d30 and d90 from 52 participants. Anti-SARS-CoV-2 spike antibodies of the IgG, IgA and SIgA isotypes were detected by ELISA. SARS-CoV-2 RNA copies were quantified by RT-PCR. SARS-CoV-2 RNA copies became negative by d14 in most subjects. At d14, 5/18 SOF samples continued to be RT-PCR-positive. Serum/SOF IgG antibodies were detected in all patients; IgG/IgA/SIgA antibodies increased by d14 and decreased by d90. At d0 and d14, SOF RNA copies were negatively correlated with SOF/serum IgG and with SOF IgA/SIgA antibodies. Higher SOF antibodies were significantly associated with a more rapid decline in SARS-CoV-2 burden. SARS-CoV-2 RNA copies and spike-specific antibodies showed differential trends during the major and minor COVID-19 waves in India. Avidity indices for SOF IgG/IgA antibodies declined by d90. Taken together, our data suggest a potential functional role for SOF anti-SARS-CoV-2 spike antibodies in clearing SARS-CoV-2 from the oral mucosa.

## 1. Introduction

SARS-CoV-2 is a coronavirus that primarily infects aerodigestive mucosal epithelial cells. The mucosal secretions of the oral cavity and the upper respiratory tract contain many anti-viral factors (innate and adaptive) that contribute to the immediate defense against invading pathogens [1,2]. IgA and secretory IgA (SIgA) antibodies play a major role in a variety of mucosal viral infections [3,4]. The presence of these anti-viral antibodies helps in confining the virus to the upper respiratory tract and mediates the subsequent clearance of the virus [5,6]. Studies have shown the presence of antigen-specific cells in germinal centers [7], lymphoid follicles [8] and dendritic cells in the oral and/or nasal mucosa, which were responsible for eliciting these IgA antibodies against SARS-CoV-2 [9,10,11].

Past exposures to SARS-CoV-2 natural infection or vaccination generally elicit a robust serum antibody response. Several studies by us and others have shown a robust humoral response to SARS-CoV-2 spike proteins in the peripheral circulation [12,13,14]. Other studies using in vitro antigen stimulation experiments have shown that the T cells of individuals exposed to SARS-CoV-2 have recall memory [15,16,17]. Both serum and mucosal anti-SARS-CoV-2 antibodies are capable of neutralizing the virus [14,18,19,20,21,22]. We and others have shown that people who encounter breakthrough infections after COVID-19 vaccinations or subsequent re-infections generally experience lesser disease severity [12,23,24].

Several studies have suggested that mucosal antibodies could play a role in the earlier clearance of the virus [20,23,24,25,26]. However, although these studies have detected mucosal antibodies in relation to SARS-CoV-2 infection, very few have determined the presence of secretory IgA (SIgA) antibodies in SOF. We previously showed, in healthcare workers (HCWs) and people living with HIV (PLWH), that local IgA/SIgA antibodies were elicited in the oral mucosal cavity upon breakthrough infections [12,27]. In the current study, we analyzed a larger cohort with COVID-19 in order to understand the role of anti-SARS-CoV-2 spike antibodies, particularly IgA/SIgA antibodies in the oral mucosa, with regard to disease severity, temporal trends and SARS-CoV-2 variants. We also aimed to study the avidity of any IgA antibodies in the SOF in order to provide clearer insights into the potential role of the locally produced SIgA antibodies.

## 2. Materials and Methods

### 2.1. Patients and Samples

The samples were collected prospectively in VHS Institutional Ethics Committee-approved studies (proposal # VHS-IEC/60-2020, VHS-IEC/72-2021 and VHS-IEC/75-2021) after obtaining written informed consent. A total of 173 participants were recruited at the Voluntary Health Services and Tamil Nadu Government Multi Super Speciality Hospital, Chennai, India, between July 2020 and May 2023—14 non-infected vaccinated controls (NIVC), 127 patients with either asymptomatic or mild COVID-19 and 32 patients with moderate or severe COVID-19. COVID-19 diagnosis was confirmed by a positive SARS-CoV-2 RT-PCR in nasopharyngeal swabs (NPSs). Disease severity in COVID-19 patients was determined using the NIH COVID-19 severity score [28]. Peripheral blood samples and stimulated oral fluid (SOF) samples were collected as described previously [29]. From NIVC participants, samples were collected at two time points four weeks apart (d0 and d30). From COVID-19 patients, samples were collected at recruitment (d0), which was within 2–4 days of disease onset. Longitudinal samples were collected from a subset of mild COVID-19 patients at 14 days (d14), 30 days (d30) and 90 days (d90) after disease onset.

### 2.2. Anti-SARS-CoV-2 Spike Antibody and Avidity Assays

Serum from the peripheral blood samples and the supernatants from the SOF samples were processed and stored at −80 °C as described previously [12] for antibody assays. Anti-SARS-CoV-2 spike IgG antibodies (referred to henceforth as IgG antibodies) were measured using a commercial ELISA kit as per the manufacturer’s instructions (Invitrogen, MA, USA). Anti-SARS-CoV-2 spike IgA antibodies (referred to henceforth as IgA antibodies) and anti-SARS-CoV-2 spike secretory IgA antibodies (referred to henceforth as SIgA antibodies) were measured using in-house ELISAs, pooled sera standards for IgA antibodies and pooled SOF standards for SIgA antibodies [12]. Avidity assays of anti-SARS-CoV-2 spike IgG and IgA antibodies were performed using the 4M urea dissociation method, as described by us previously [27]. The avidity index is a percentage value calculated by dividing the OD value with 4M urea by the OD value without urea for each sample. If there was no dissociation, then the index remained at 100%.

### 2.3. Quantitative SARS-CoV-2 RNA Estimation

The deposits of the SOF samples were processed immediately to determine the SARS-CoV-2 RNA copies using real-time RT-PCR, as described by us previously [30,31].

### 2.4. Statistical Analysis

The secretion rates (SRs) were calculated by dividing the volume of the SOF sample by the corresponding collection time for each participant. The antibody levels in the SOF samples were analyzed as absolute values (U/mL) and normalized over their corresponding SRs (U/mL/min). The mean and median values were calculated using Microsoft Excel. Mann–Whitney rank sum tests were used to compare medians, Spearman rank correlation coefficients and their corresponding statistical significance were used to analyse the correlations between measurements at various time points; *t*-tests were used for mean value comparisons; and Fisher’s exact test was used to assess the significance of moderate COVID-19 severity between the different COVID-19 waves. Calculations were performed using GraphPad Prism version 8.0 and the free online calculator from Social Science Statistics.

## 3. Results

### 3.1. SARS-CoV-2 Burden in Stimulated Oral Fluid (SOF) from COVID-19 Patients

**Figure 1:**
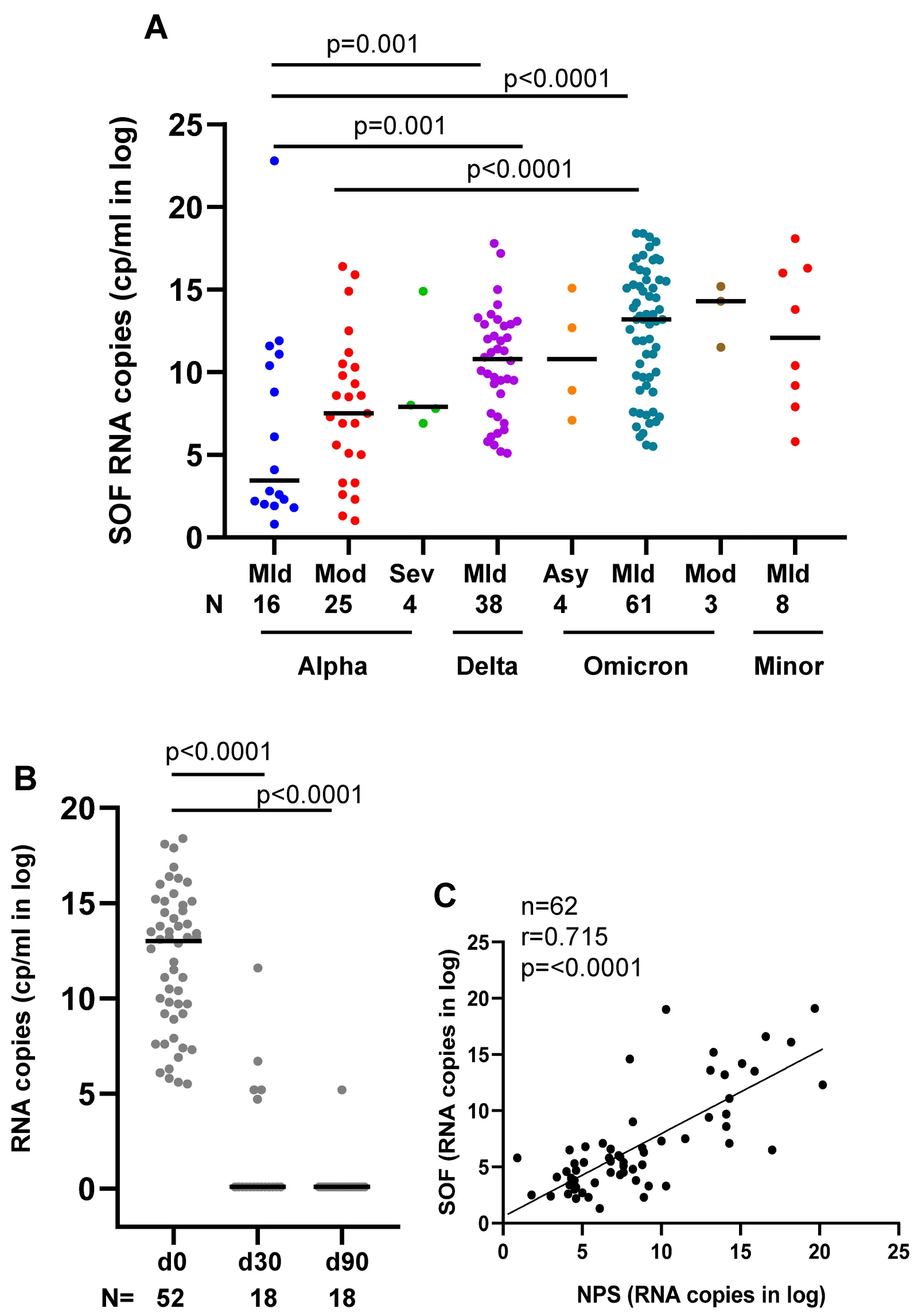
SARS-CoV-2 RNA burden in COVID-19 patients. The *X*-axis denotes the categories. The *Y*-axis denotes the SARS-CoV-2 RNA copies per milliliter in logarithmic values. The circles denote individual samples, and the short bar indicates the median value of each group. NPS = nasopharyngeal swab. (**A**) SARS-CoV-2 RNA copies in SOF among asymptomatic (Asy), mild (Mld), moderate (Mod) or severe (Sev) COVID-19 during the Alpha, Delta, Omicron and minor variant waves. (**B**) Longitudinal analysis of the SARS-CoV-2 burden in SOF samples. (**C**) Correlation analysis between NPS RNA copies and SOF RNA copies. r—correlation coefficient; *p*—*p* value (Spearman rank correlation coefficient test).

Based on the time period of sample collection, all samples were stratified according to the different COVID-19 waves, as designated by the variant SARS-CoV-2 strains that were predominant at the time of sample collection. Of the 159 COVID-19 patients, the SARS-CoV-2 RNA copy numbers in SOF samples were significantly lower when infected with the Alpha strain during the first wave in both the groups of mild or moderate disease severity (Figure 1A). The highest median RNA copies were seen with the Omicron strain (median: 13.2 cp/mL in log) during the third wave. The SARS-CoV-2 RNA load increased significantly during the Delta and Omicron waves; however, the number of cases with moderate disease severity significantly declined during the Omicron wave compared to the Alpha wave (*p* < 0.0001; Fisher exact test; Figure 1A). Longitudinal SOF samples were available from a subset of the COVID-19 patients during the Omicron and minor waves. The RNA copies in SOF declined from a median log of 13 cp/mL at onset (d0) to almost negative by d14 in the majority of patients. Only 5/18 (28%) and 1/18 (6%) remained positive for RNA at d14 and d30 after disease onset (Figure 1B).

The SARS-CoV-2 RNA copy numbers were compared between the NPS and SOF samples collected concomitantly from 62 patients. There was a strong positive correlation between the two sample types (*p* < 0.0001; Spearman rank correlation coefficient test; Figure 1C). These findings suggest that the SARS-CoV-2 RNA burden in the SOF was low in patients during the Alpha wave with moderate/severe disease, while the burden was higher in patients with mild disease during the Delta or Omicron waves, which were more infective in nature.

### 3.2. Differential Expression of Salivary Anti-SARS-CoV-2 Spike Antibodies

**Figure 2:**
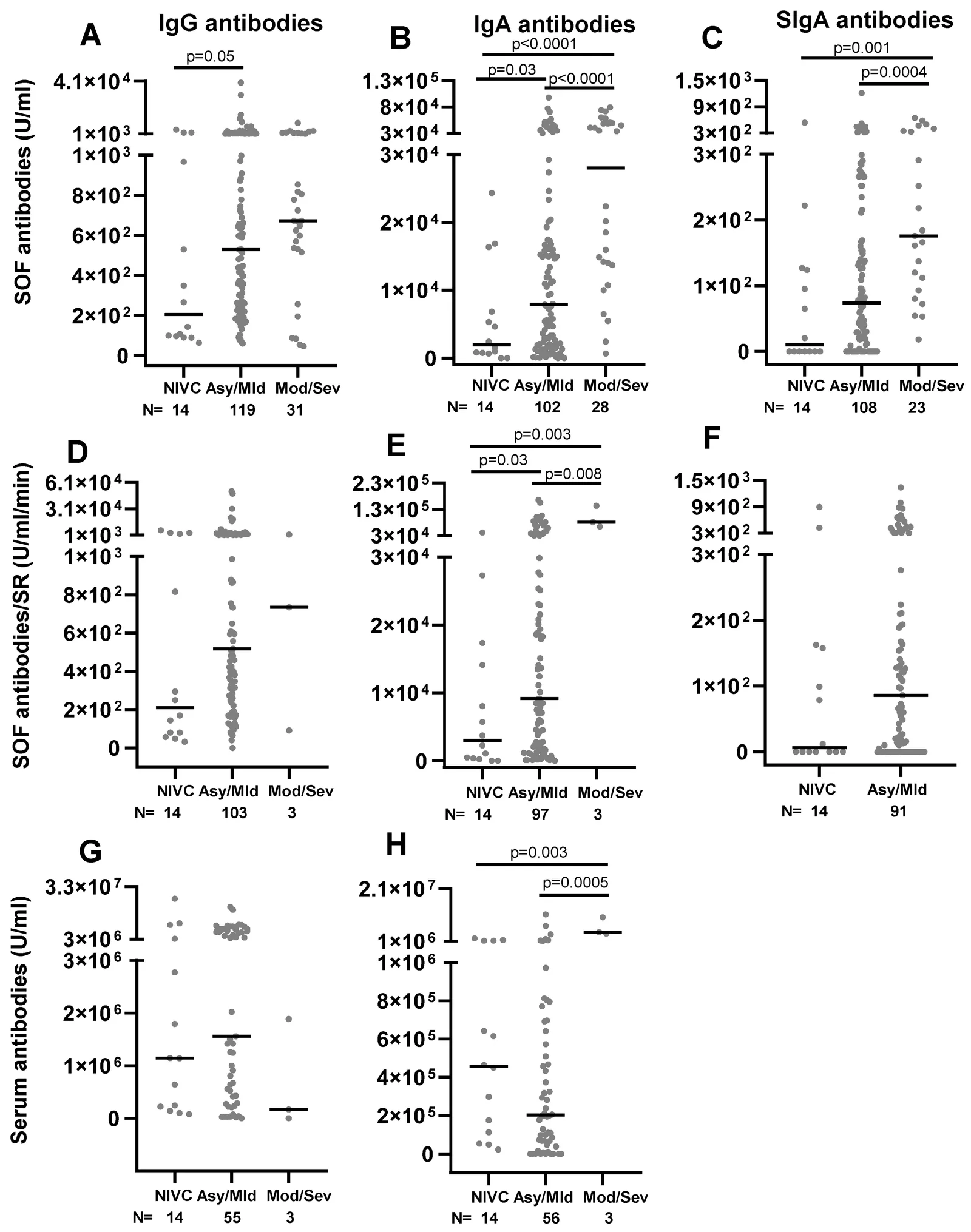
Anti-SARS-CoV-2 spike antibodies in COVID-19 patients with asymptomatic/mild or moderate/severe disease. The *X*-axis denotes the COVID-19 disease severity categories. The *Y*-axis denotes the anti-SARS-CoV-2 spike antibodies. The circles denote individual samples, and the short bar indicates the median value of each group. The graphs of the first column denote IgG antibodies (panel (**A**) as absolute values in SOF; panel (**D**) as values normalized over the SR in SOF; panel (**G**) as values in serum). The graphs in the second column denote IgA antibodies (panel (**B**) as absolute values in SOF; panel (**E**) as values normalized over the SR in SOF; panel (**H**) as values in serum). The graphs in the third column denote SIgA antibodies (panel (**C**) as absolute values in SOF; panel (**F**) as values normalized over the SR in SOF).

In the SOF samples, the IgG antibodies were markedly lower in NIVC (median: 205 U/mL) than in COVID-19 patients (*p* = 0.05; Figure 2A). The IgG antibodies were lower in patients with asymptomatic or mild COVID-19 (median: 529 U/mL) compared to patients with moderate or severe COVID-19 (median: 673 U/mL). The trends of IgA antibodies and SIgA antibodies in the SOF samples were similar to those of the IgG antibodies (Figure 2B,C, respectively). These antibody levels were also analyzed after normalizing them over the salivary secretion rates (SRs) correspondingly (Figure 2D–F). These normalized antibody levels also showed the same trends among the NIVC and COVID-19 cohorts as for the antibody levels without normalization (comparing Figure 2A–C with Figure 2D–F, respectively). This suggests that a robust anti-SARS-CoV-2 spike antibody response was elicited in the oral mucosa upon acquiring COVID-19.

The IgG (Figure 2G) and IgA (Figure 2H) antibodies in the concomitant serum samples of a subset showed a slightly different trend from the SOF samples (Figure 2A,B, respectively). Patients with moderate/severe disease elicited lower IgG antibodies (median: 168,200 U/mL) and higher IgA antibodies (median: 4,464,233 U/mL) compared with patients with asymptomatic or mild disease (IgG antibody median: 1,560,800 U/mL; IgA antibody median: 202,700 U/mL). Thus, there was differential expression of IgG and IgA antibodies in the SOF and serum samples among COVID-19 patients with different disease severities.

### 3.3. Inverse Correlation Between SARS-CoV-2 RNA Copies and Anti-SARS-CoV-2 Spike Antibodies

**Figure 3:**
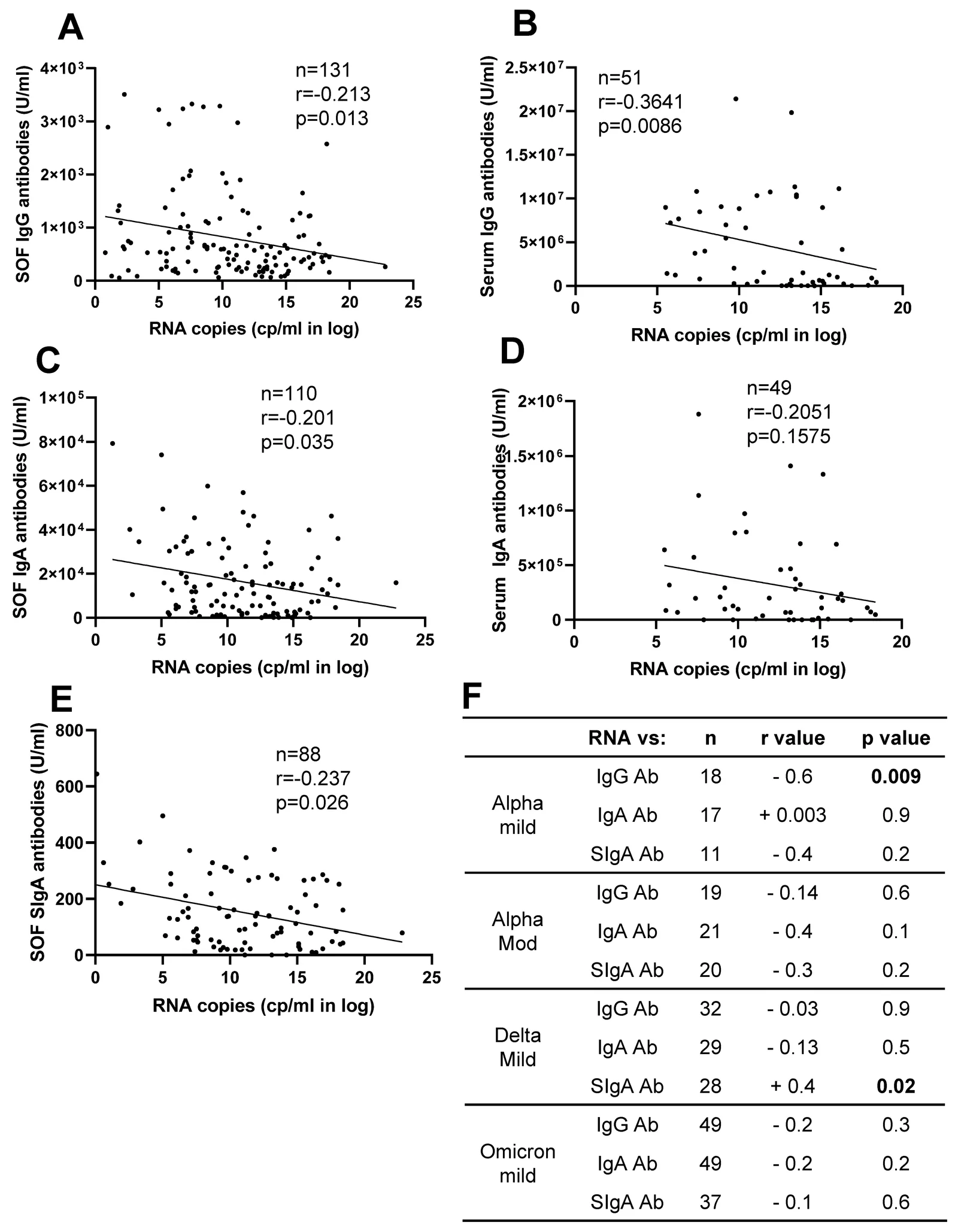
Correlations between SARS-CoV-2 RNA copies and anti-SARS-CoV-2 antibodies. The *X*-axis denotes the SARS-CoV-2 RNA copies/mL in logarithmic values. The *Y*-axis denotes the antibody levels in SOF or serum. The circles denote each sample. (**A**) SOF IgG antibodies in U/mL. (**B**) Serum IgG antibodies in U/mL. (**C**) SOF IgA antibodies in U/mL. (**D**) Serum IgA antibodies in U/mL. (**E**) SOF SIgA antibodies in U/mL. (**F**) Table inset showing the Spearman correlation coefficients comparing the SOF RNA copies and the SOF anti-SARS-CoV-2 antibodies stratified by the SARS-CoV-2 variant. r—correlation coefficient; *p*—*p* value. The r and *p* values were calculated using the Spearman rank correlation test.

The SARS-CoV-2 RNA copies in the SOF samples showed an inverse correlation against the IgG antibodies (r = −0.213; Figure 3A), IgA antibodies (r = −0.201; Figure 3C) and SIgA antibodies (r = −0.237; Figure 3E) in the SOF samples as well as the serum samples (IgG: r = −0.364, Figure 3B; IgA: r = −0.205, Figure 3D). All these Spearman rank correlations reached statistical significance, except that for serum IgA antibodies. The correlations between the RNA copies of the virus and the anti-SARS-CoV-2 specific antibodies during the different COVID-19 waves showed similar negative correlations (Figure 3F), except for the Delta variant. During the Delta variant wave, the SOF RNA copies showed a positive correlation with the anti-SARS-CoV-2 spike SIgA antibodies. The overall negative correlations show that higher IgG, IgA and SIgA antibodies are associated with lower mucosal viral counts and virus-specific antibodies.

### 3.4. Avidity Indices of IgG and IgA Antibodies

We analyzed the technical reproducibility of the avidity assays by performing the experiments at two independent times. The mean CV% of the IgG antibody avidity index was 3.3% in serum (n = 80) and 3.1% in SOF (n = 12). The mean CV% of the IgA antibody avidity index was 4.3% in serum (n = 15). This suggests that the optimized avidity assays for the IgG and IgA antibodies in both serum and SOF samples were reproducible and reliable.

**Figure 4:**
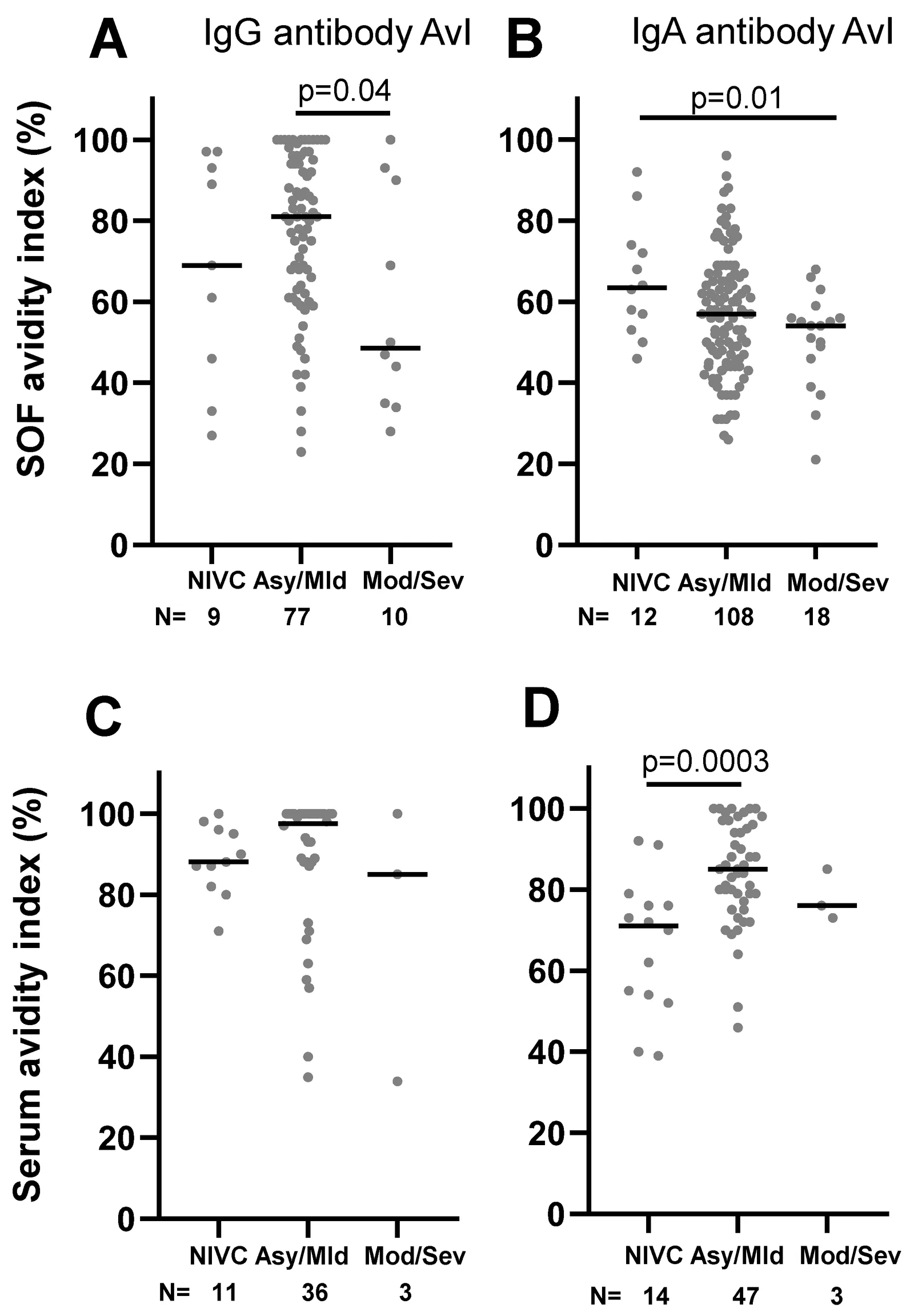
Avidity indices (AvI) of IgG and IgA antibodies in SOF and serum. The *X*-axis denotes the COVID-19 severity categories. The *Y*-axis denotes the avidity indices as percentages. The circles denote the samples, and the short bar denotes the median values. (**A**) IgG antibody avidity in SOF; (**B**) IgA antibody avidity in SOF; (**C**) IgG antibody avidity in serum; (**D**) IgA antibody avidity in serum.

The avidity indices of IgG antibodies in the SOF (Figure 4A) and serum (Figure 4C) samples followed a similar trend between the NIVC and COVID-19 cohorts, although the avidity indices were higher in the serum compared with the SOF. The median IgG antibody avidity was lower in the SOF of those who developed moderate/severe disease compared to those with milder disease. The SOF IgA antibody avidity indices in those who developed COVID-19 were lower than in the NIVC (Figure 4B), while they showed the opposite trend in the serum (Figure 4D). In moderate/severe COVID-19 patients, although the antibody levels were high, their avidity was low, suggesting a poorer neutralization capacity and, in turn, poorer viral clearance.

### 3.5. Longitudinal Trends of Anti-SARS-CoV-2 Spike Antibodies and Avidity Indices

**Figure 5:**
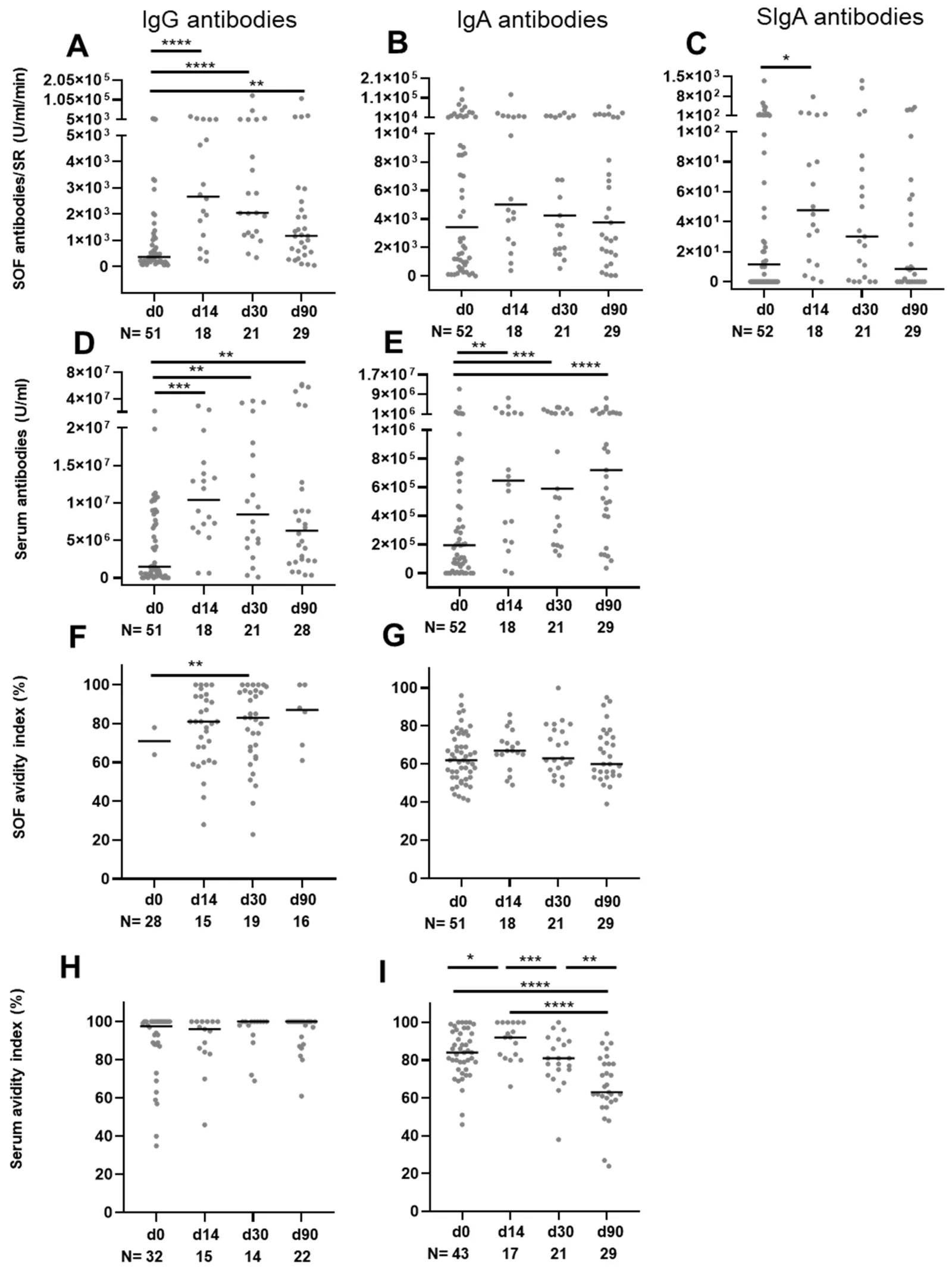
Longitudinal trends of IgG, IgA and SIgA antibodies and their avidity indices in SOF and serum samples. The *X*-axis denotes the sample collection time point in days. The *Y*-axis denotes the antibody levels or avidity indices. The bars denote the median values. (**A**) SOF IgG antibodies normalized over the SR (U/mL/min). (**B**) SOF IgA antibodies normalized over the SR (U/mL/min). (**C**) SOF SIgA antibodies normalized over the SR (U/mL/min). (**D**) Serum IgG antibodies (u/mL). (**E**) Serum IgA antibodies (U/mL). (**F**) SOF IgG antibody avidity index (%). (**G**) SOF IgA antibody avidity index (%). (**H**) Serum IgG antibody avidity index (%). (**I**) Serum IgA antibody avidity index (%). *p* values were calculated by Mann–Whitney rank sum test. *: *p* < 0.05; **: *p* < 0.01; ***: *p* < 0.001; ****: *p* < 0.0001.

In mild COVID-19 patients, there was a significant increase in the IgG (Figure 5A) and SIgA (Figure 5C) antibody levels in the SOF at d14 after disease onset. Although the IgA antibodies (Figure 5B) in the SOF also showed a similar trend, it was not statistically significant. Similar rises in serum IgG (Figure 5D) and IgA (Figure 5E) antibodies showed statistical significance. By d30 and d90 after disease onset, the IgG, IgA and SIgA antibodies declined in both SOF and serum samples. However, at d90, the levels of SOF IgG antibodies, serum IgG antibodies and serum IgA antibodies were still significantly higher than at disease onset. While, SOF IgA/SR antibodies declined to the same level as at d0, and SOF SIgA/SR declined below the levels of d0. This suggests a robust mucosal antibody response within d14 of disease onset and indicates that SOF IgA and SIgA antibodies are relatively short-lived.

The median avidity indices of IgG (Figure 5F) antibodies in the SOF showed an increase from disease onset, but there was no obvious change for SOF IgA (Figure 5G). The median IgG antibody avidity index at d30 in SOF was statistically higher than at d0 (*p* = 0.007). By d90, the SOF IgG antibody avidity index began to decline, while, in the serum, the IgG antibody avidity indices (Figure 5H) remained high. The serum IgA antibody avidity indices declined by d30 and were statistically lower than at all subsequent time points (Figure 5I). Thus, the serum IgA antibodies were less avid and declined more rapidly than the serum IgG antibodies.

### 3.6. IgG, IgA and SIgA Antibody Responses Elicited by Different SARS-CoV-2 Strains

**Figure 6:**
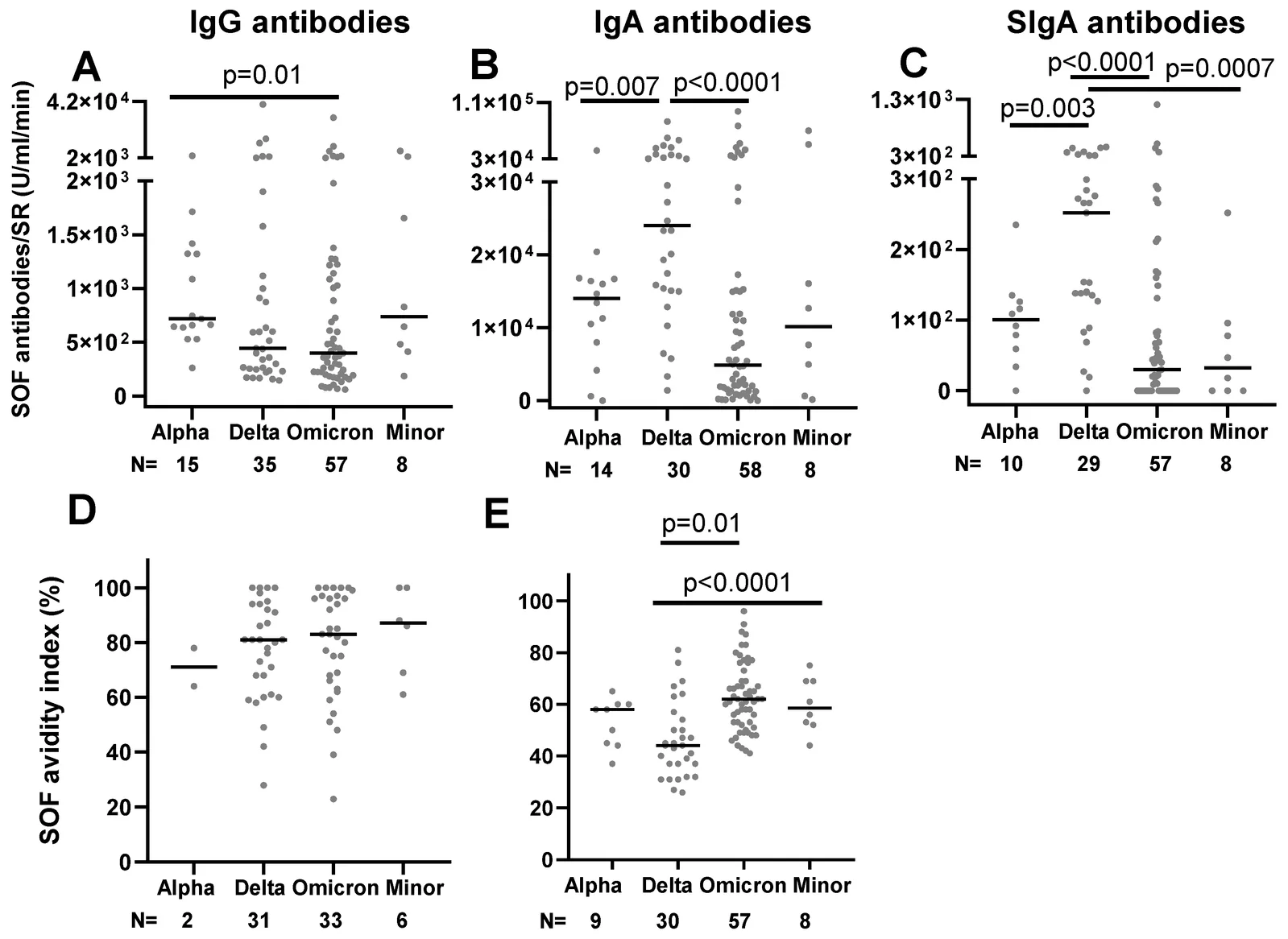
Anti-SARS-CoV-2 spike IgG, IgA and SIgA antibodies and their avidity indices in SOF samples from COVID-19 patients during different COVID-19 waves. The *Y*-axis denotes the antibodies or avidity index. The short bars indicate the median values. (**A**) SOF IgG antibodies normalized over SR (U/mL/min). (**B**) SOF IgA antibodies normalized over SR (U/mL/min). (**C**) SOF SIgA antibodies normalized over SR (U/mL/min). (**D**) SOF IgG antibody avidity indices (%). (**E**) SOF IgA antibody avidity indices (%). *p* values were calculated by Mann–Whitney rank sum test.

We next analyzed the IgG, IgA and SIgA antibody levels and their avidity indices in the SOF samples by stratifying them according to the prevalent SARS-CoV-2 variant (Figure 6). The levels of SOF IgA and SIgA antibodies elicited by the Alpha strain were significantly lower than those elicited by the Delta strain (IgA antibodies: *p* = 0.007; SIgA antibodies: *p* = 0.003) but higher than for the Omicron strain (IgA antibodies: *p* < 0.0001; SIgA antibodies: *p* < 0.0001). On the other hand, the Alpha strain elicited the highest levels of SOF IgG antibodies (Figure 6A).

The IgG antibody avidity indices showed an increasing trend with every subsequent COVID-19 wave (Figure 6D), as expected. However, the SOF IgA avidity index (Figure 6E) was significantly lower against the SARS-CoV-2 Delta strain compared to the Omicron strain (*p* = 0.01) and the minor waves (*p* < 0.0001). Thus, the IgG antibodies were more avid even though they were lower in concentration, while the trends of the IgA antibodies and their avidity indices were variable.

### 3.7. Prior SARS-CoV-2 Exposure Facilitates Earlier Clearance of the Virus

**Figure 7:**
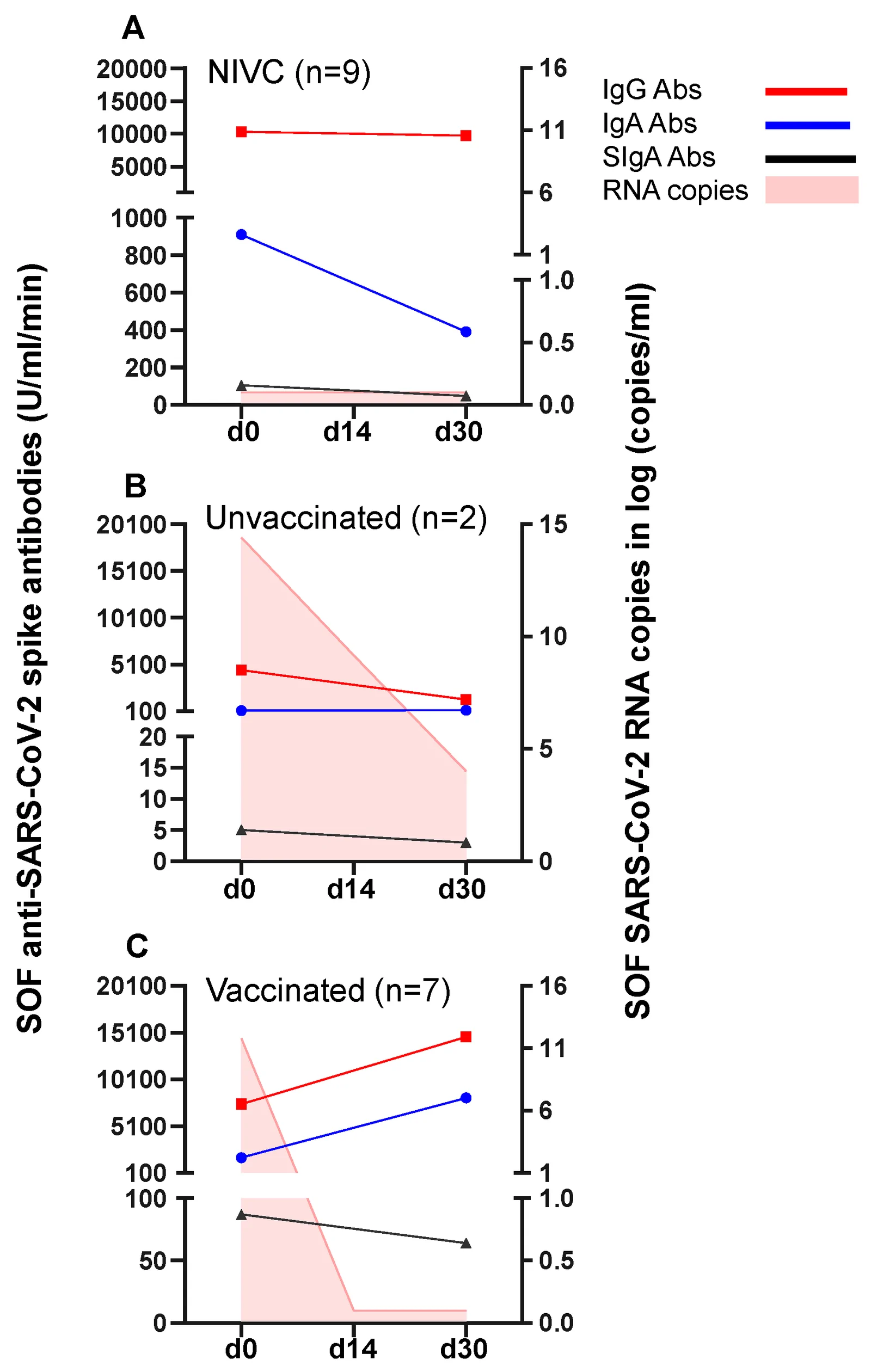
Differential trends in SOF SARS-CoV-2 burden and SOF anti-SARS-CoV-2 spike antibodies normalized over the secretion rate (SR) among unvaccinated or vaccinated mild COVID-19 patients. The *X*-axis denotes the sample time point; the pink shaded area denotes the mean SARS-CoV-2 RNA copies/mL in SOF. Red—mean anti-SARS-CoV-2 spike IgG/SR antibodies; blue—mean anti-SARS-CoV-2 spike IgA/SR antibodies; black—mean anti-SARS-CoV-2 spike SIgA/SR antibodies. (**A**) Non-infected vaccinated controls (NIVC). (**B**) Unvaccinated COVID-19 patients. (**C**) Vaccinated COVID-19 patients.

Of the 52 mild COVID-19 patients from whom longitudinal SOF samples were available, two were unvaccinated and recruited during the Omicron wave. Seven mild COVID-19 patients who developed breakthrough infections and were part of a previous COVID-19 vaccine trial were recruited in this study during the same Omicron wave. Nine NIVC participants recruited during the Omicron wave were also included for this analysis. The SARS-CoV-2 RNA burden became negative by d14 in the vaccinated group but remained positive till d30 in the unvaccinated group (Figure 7). The SOF IgG/SR, IgA/SR and SIgA/SR antibodies showed a declining trend in the NIVC group and declining or no antibody elicitation in the unvaccinated group but an increasing trend in the vaccinated group.

Taken together, the findings of this study suggest that the immediate elicitation of IgG, IgA and SIgA antibodies in the SOF due to prior exposure from vaccination or previous infection facilitated the earlier clearance of SARS-CoV-2.

## 4. Discussion

A virus-induced inflammatory response can sometimes cause higher morbidity than the virus infection itself due to the cytolytic nature of the elicited host immune response. In this study, we showed that anti-SARS-CoV-2 spike IgG, IgA and SIgA antibodies were detectable soon after the onset of COVID-19 in the oral cavity, which is a portal of entry for SARS-CoV-2. We also showed inverse trends and correlations between the SARS-CoV-2-specific mucosal IgA/SIgA antibodies and the SARS-CoV-2 RNA burden, thereby suggesting a role in the earlier clearance of SARS-CoV-2 from the mucosae. This, in turn, might help in reducing the local inflammatory response, which would reduce tissue injury-mediated morbidity/disease severity.

SOF is a non-invasive and easy-to-collect specimen that represents the mucosae of the aerodigestive tract; hence, SOF is an ideal specimen to study the infectious agents and host factors that are involved in respiratory and gastrointestinal infectious and non-infectious inflammatory conditions [3,4]. Saliva is another easy-to-collect and non-invasive sample that has been validated as a diagnostic sample for the detection of respiratory viruses [32,33,34]. However, there are a number of challenges and limitations faced in analyzing the mucosal antibodies in SOF with accuracy due to variations in the collection and the type of SOF sample; the detection of the secretory component of the IgA antibody; and normalization for physiological differences like protein content, the salivary SR and the total isotype-specific antibody production [35]. In our study, SOF was collected by the stimulation of chewing with inert paraffin wax. This method of collection includes the oral secretions from all salivary glands (major and minor) and also the gingival crevicular fluid. The SIgA antibody measurements were performed using ELISA and an anti-human secretory IgA monoclonal antibody. Additionally, we compared our SOF antibody data with both actual values and antibody values normalized over their corresponding salivary SRs. Taken together, we can conclude that our data are accurate and highly representative of the mucosal antibodies that were locally produced and are hence of direct clinical relevance.

The SARS-CoV-2 RNA copy numbers were statistically higher in the mild disease groups during the Delta and Omicron variant waves when compared with the mild disease group during the Alpha variant wave. It is important to note that we had greater numbers of moderate/severe cases during the first Alpha variant wave (29/45—64%) than during the second Delta variant wave (0/38), which was soon after the initiation of the vaccination program, and the third Omicron variant wave (3/68—4%), which was more than six months after the initiation of vaccination, when the anti-SARS-CoV-2-specific antibodies began to decline. It is known that the rates of infectivity and transmissibility were higher during the Delta and Omicron waves when compared with the Alpha wave [31,36]. Taken together, the data suggest that the presence of mucosal antibodies (Figure 2) and B cell memory [12,13,17,37] in SARS-CoV-2 might have facilitated lower disease severity via the earlier clearance of the virus (as shown in Figure 7).

In moderate or severe COVID-19, the IgG, IgA and SIgA antibodies in the SOF were higher than in mild disease. This suggests the greater elicitation of a humoral immune response. In the peripheral circulation, the IgG antibodies were lower in moderate or severe disease. This suggests an increase in the passive movement of IgG antibodies into the mucosal compartment from the peripheral circulation. The higher total IgA antibodies and the dimeric secretory IgA antibodies in the SOF suggest the robust local elicitation of a humoral immune response. The IgG antibodies in the SOF were orders of magnitude lower than the levels in the serum. On the other hand, the IgA antibodies in the SOF were only one order of magnitude lower than the serum antibodies. This is suggestive of local production in addition to passive movement. Sterlin et al. have shown that IgA plasmablasts increase in the blood during COVID-19 and circulate longer compared to IgG plasmablasts [38].

The anti-SARS-CoV-2 spike-specific IgG, IgA and SIgA antibodies and the SARS-CoV-2 RNA burden showed statistically significant inverse correlations. Although the statistical significance diminished when these analyses were stratified by the different SARS-CoV-2 variants, the inverse trend remained, suggesting a contrasting association between the virus and the humoral immune response. During the Delta variant wave, there was a positive correlation between the RNA copies and the SIgA antibody levels. This most likely reflects the higher circulating levels of anti-SARS-CoV-2 antibodies post-vaccination. Longitudinal analyses showed that the SARS-CoV-2 RNA burden became negative by d14 in most of the mild COVID-19 patients. During these 14 days from disease onset, the IgG, IgA and SIgA antibodies were present in significantly high levels in both SOF and serum samples. Additionally, the SARS-CoV-2 RNA copies continued to persist in the oral mucosa even at d30 in the two unvaccinated individuals, who also had very low anti-SARS-CoV-2 spike antibody levels, when compared with the vaccinated individuals, in whom the virus-specific antibody levels were higher and the RNA copies were undetectable by d14. Although the patient numbers were very small, these findings, taken together with the negative correlations between the antibodies in SOF and the RNA copy numbers, strongly suggest that the early elicitation of the mucosal antibody response against SARS-CoV-2 (probably due to vaccination in this case) facilitates the clearance of the virus.

We have not demonstrated neutralizing activity of antibodies in SOF in this study. Neutralizing activity of antibodies in saliva has been shown [23,39,40], but isotype specificity was not demonstrated. In the latter study [40], vaccine-elicited saliva antibodies were able to block the binding of hACE-2 to the spike RBD of the reference strain. In a murine model, induced SIgA antibodies were shown to have neutralizing activity [41]. A direct link between mucosal IgA and protection against influenza virus infection in humans has been demonstrated [42]. These findings support the view that both IgG and SIgA antibodies in mucosal secretions can contribute to the clearance of viruses. This is reiterated by many other studies that have shown the neutralization capacity of these antibodies [19,20,21,22,38,43], as well as inverse correlations with the SARS-CoV-2 RNA load and disease severity [6,9,10,37,44].

The avidity of antibodies indicate the strength of their binding to the corresponding antigens, which in turn reflects their neutralization capacity. In our study, the avidity of both the IgG and IgA antibodies was lower in the SOF compared to the serum and was lower in patients with moderate or severe COVID-19 compared to those with mild COVID-19. This could also be attributed to the delayed clearance of the virus, which in turn could lead to higher morbidity and hence higher disease severity. Avidity increases during convalescence and also upon repeated exposures to the same antigen [45]. Among the mild COVID-19 patients, the longitudinal analysis of the IgG antibody avidity indices showed an increase with time in both SOF and serum samples. The IgG antibodies seen in the oral cavity were derived primarily from the peripheral circulation. On the other hand, the avidity indices of the IgA antibodies declined by d30 and d90. This was likely due to the shorter half-life of IgA antibodies [38]. This decline was more rapid in the serum, in contrast to the slower decline in the SOF. The slower decline in the IgA antibody avidity indices could be attributed to the persistent higher SOF IgA levels at d90. IgG antibody avidity continued to remain high even though the IgG antibody levels declined. This suggests the potential selection of plasma cells that secrete more avid antibodies upon convalescence in order to retain immunological memory for future exposures.

In India, there were three major waves and a few minor waves of COVID-19 from March 2020 to late 2023. We collected SOF samples during various periods of these COVID-19 waves as part of three different studies. Therefore, the SARS-CoV-2 burden and the anti-SARS-CoV-2 antibodies analyzed in these SOF samples were stratified based on the SARS-CoV-2 strain type that was predominant during the corresponding sample collection period. The SARS-CoV-2 RNA burden was highest during the Omicron wave and lowest during the first Alpha wave. This indicates the infectivity and transmissibility of these strain types. During the early pandemic, SARS-CoV-2 involvement was greater in the lungs and required the testing of nasopharyngeal swabs. By the time that the Omicron variant became prevalent, the disease was more confined to the upper respiratory tract. Earlier studies were able to detect SARS-CoV-2 in the saliva only in about 80% of cases confirmed by nasopharyngeal swabs [30,31]. During the subsequent waves, SARS-CoV-2 was detected in 100% of saliva samples, making self-testing with saliva or nasal swabs at home more convenient and reliable.

The Delta strain elicited the highest levels and the Omicron strain elicited the lowest levels of IgA/SIgA antibodies. Studies have shown that the antibodies elicited against the Omicron strain were minimally detectable by assays using the ancestral strain spike RBD due to antigenic drift in the spike protein of the Omicron variant; however, the antibodies were cross-protective [46,47,48]. The mild nature of the disease among the previously vaccinated patients recruited during the Omicron wave, their quick clearance of the virus within 14 days, and their increasing antibody avidity seen in our study are all suggestive of a strong mucosal immune response that could potentially contribute to immunological imprinting [12,25,46,47,48,49,50,51,52,53].

COVID-19 vaccination programs was notably effective in reducing disease severity. We had previously shown the robust elicitation of IgG, IgA and SIgA antibodies in serum and SOF samples from HCWs and PLWH post-COVID-19 vaccination [12,27]. In the current study, we further showed that the number of patients with moderate or severe disease (3/72; 4.2%) post-COVID-19 vaccination (Omicron and minor waves) was significantly lower than during the first wave (29/45; 64%). During the third wave, there was a small group of two mild COVID-19 patients who did not receive the COVID-19 vaccine. Longitudinal analyses in a small comparative subset showed that the vaccinated patients with BTI cleared the virus within 14 days, while unvaccinated patients continued to carry a higher SARS-CoV-2 burden in the SOF at d14. The IgG, IgA and SIgA antibodies showed a rising trend at d30 in the vaccinated group but a declining trend in the NIVC group. In the unvaccinated group, the IgG and SIgA antibody levels in the SOF were negligible, while the IgA antibodies showed a declining trend in one of the two patients. Since vaccination was mandated during the COVID-19 pandemic, the small number of unvaccinated patients posed a statistical limitation; however, the varying trends clearly indicate the benefit of vaccination.

## 5. Conclusions

In this study, we have shown the differential trends of the SARS-CoV-2 burden and the anti-SARS-CoV-2 spike antibodies for the first time during the major and minor COVID-19 waves in India. The stronger IgA antibody avidity in the SOF, shown in this study for the first time, confirms the local SIgA antibody production in the SOF in COVID-19. This research demonstrates an inverse relationship between salivary antibodies and the SARS-CoV2 burden, suggesting the potential relevance of mucosal antibodies in the protection against viral infections of all mucosal surfaces.

## Data Availability

All the data are given in this article.
